# EXPLORATION OF STRESS FLUCTUATIONS IN FAMILY CAREGIVERS OF OLDER PEOPLE WITH MILD COGNITIVE IMPAIRMENT

**DOI:** 10.1093/geroni/igad104.3328

**Published:** 2023-12-21

**Authors:** Sonoko Kabaya, Chieko Greiner, Masahide Nakamura, Yuko Yamaguchi, Hirochika Ryuno

**Affiliations:** Kobe University, Kobe-city, Hyogo, Japan; Kobe University, Kobe-city, Hyogo, Japan; Kobe University, Kobe-city, Hyogo, Japan; Kobe University, Kobe-city, Hyogo, Japan; Kobe University, Kobe-city, Hyogo, Japan

## Abstract

Mild cognitive impairment (MCI) is common, affecting 10%-35% of people over 65, and around two-thirds overall develop dementia over their lifetime. Previous studies have reported that family caregivers (FCs) caring for older people with MCI experience considerable burden, which tends to cause various neuropsychiatric symptoms, such as depression, apathy, and irritability. However, it is not clear how FCs’ stress, which leads to increased burden, fluctuates within a day through their daily activities. The aim of this study was to explore the characteristics of stress variability in FCs. We recruited one dyad of an older adult with MCI over the age of 65 and a FC. Data collection was performed continuously for 7 consecutive days. We adopted smart watches (GARMIN vívosmart 4) to measure Heart Rate Variability (HRV)-based stress. Moreover, we asked a FC to talk with a Virtual Agent (VA) on a laptop freely to understand when and what kind of daily activities were done and the FC’s feelings. Detecting change points in time series data was performed using Python 3.9. Results showed that change points in the FC’s daily stress appeared when the FC starts specific activities such as preparing meals and confirming some medicine that an older adult with MCI should take and daily schedules. The FC also expressed anticipatory grief at the prospect of future dementia through the VA. This finding is beneficial for considering how to reduce FCs’ stress during daily activities.

